# Supplementary figures and images for: Verity plots: A novel method of visualizing reliability assessments of artificial intelligence methods in quantitative cardiovascular magnetic resonance
Source: PLoS One. 2025 May 16;20(5):e0323371. doi: 10.1371/journal.pone.0323371 (PMC12084058; doi:10.1371/journal.pone.0323371)

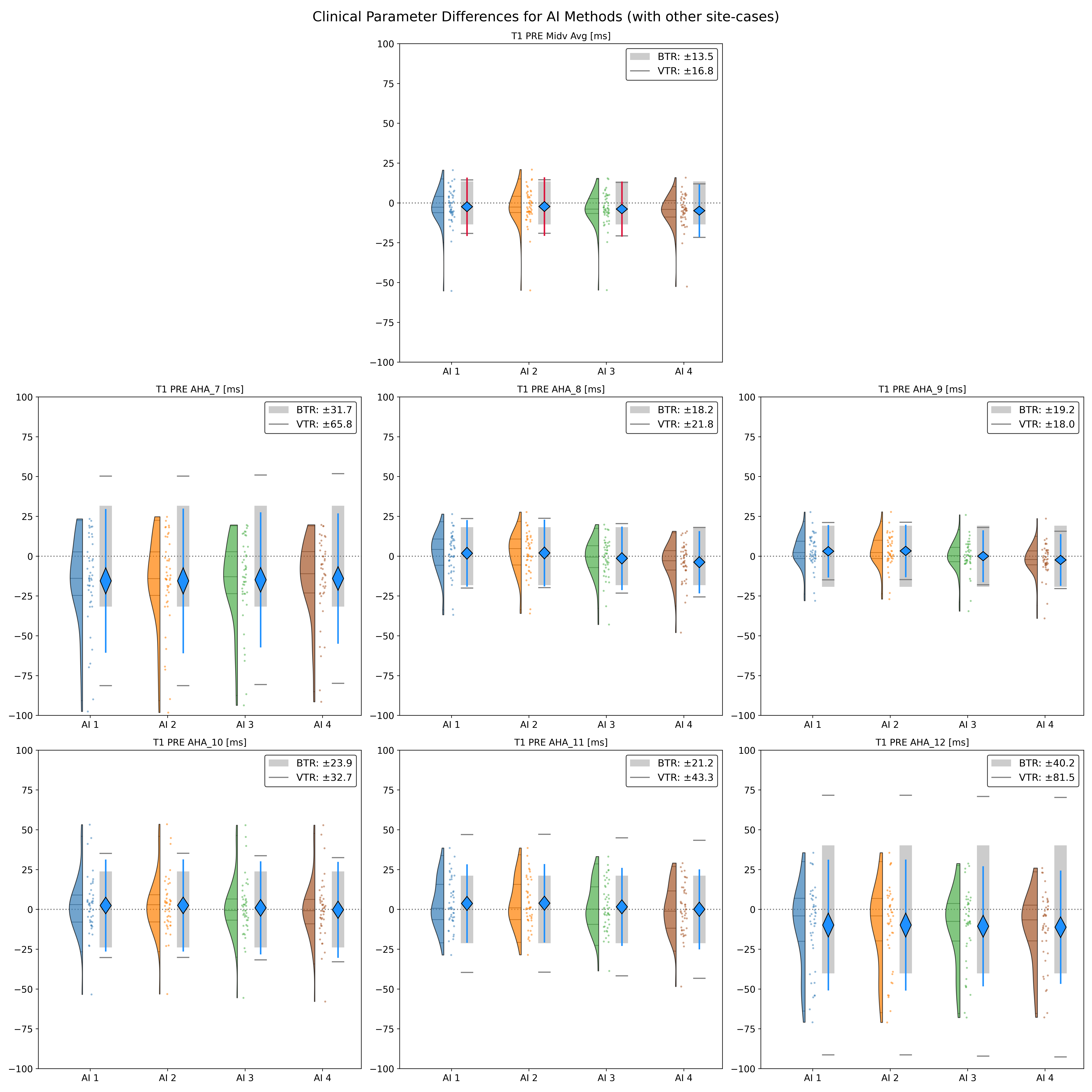

Supplement: S3_Fig — For all four AI methods, and for cases from all available sites, Condensed Verity Plots are visualized to communicate AI agreement with expert. (PNG) [file pone.0323371.s003.png]

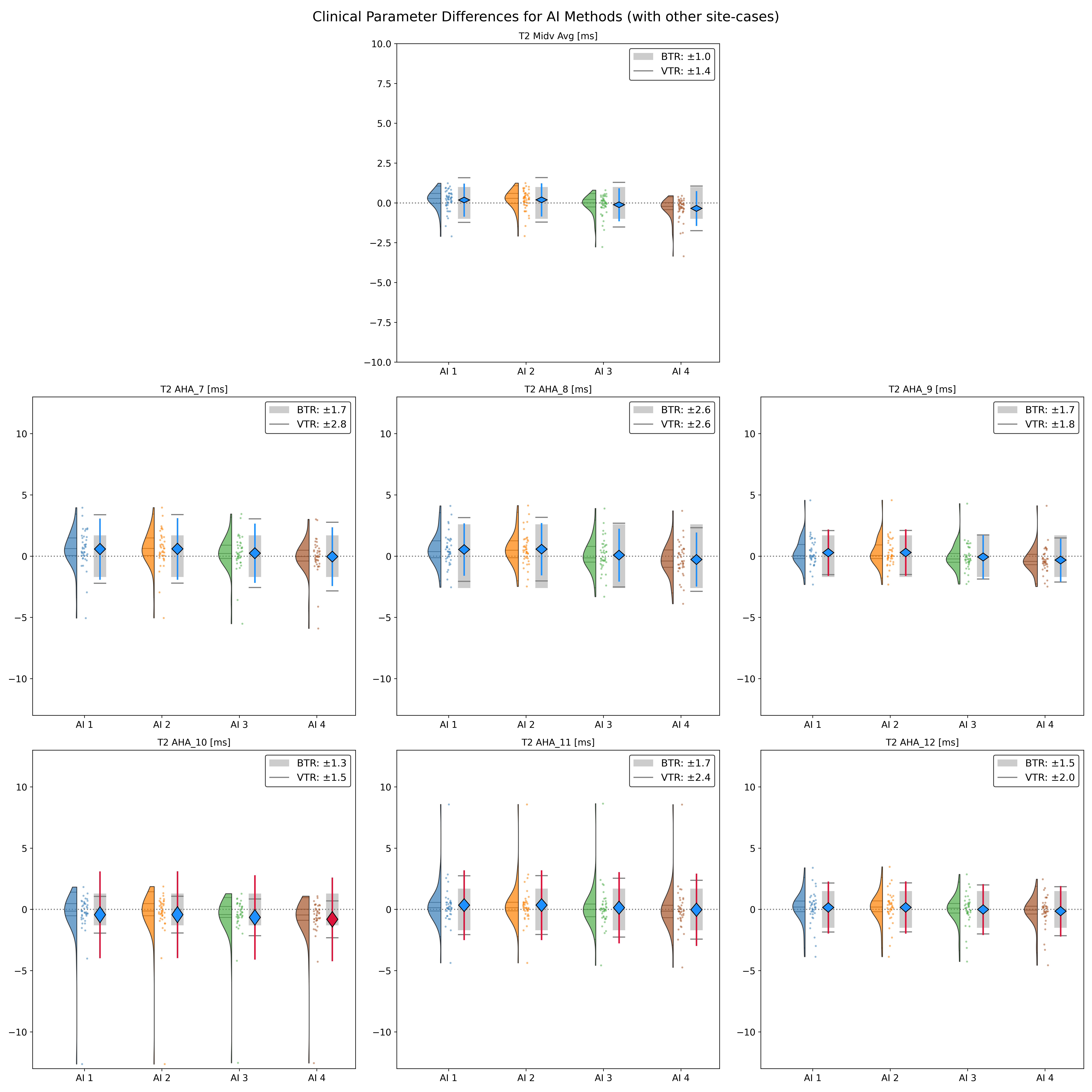

Supplement: S4_Fig — For all four AI methods, and for cases from all available sites, Condensed Verity Plots are visualized to communicate AI agreement with expert. (PNG) [file pone.0323371.s004.png]
