# Supplementary material for: Verity plots: A novel method of visualizing reliability assessments of artificial intelligence methods in quantitative cardiovascular magnetic resonance
Source: PLoS One. 2025 May 16;20(5):e0323371. doi: 10.1371/journal.pone.0323371 (PMC12084058; doi:10.1371/journal.pone.0323371)
Supplement: S1_File — AI Architecture and Training Pipeline. Describes the U-Net architecture details employed for training, as well as details of the training procedures, including image normalization and augmentation strategies. (PDF) [file pone.0323371.s005.pdf]

Supplementary Material: AI Model Architectures and Training Procedures

1) Architecture

Building on a previous scientific endeavour, in which convolutional neural network (CNN) architectures were analyzed, we implemented a U-Net [1]:

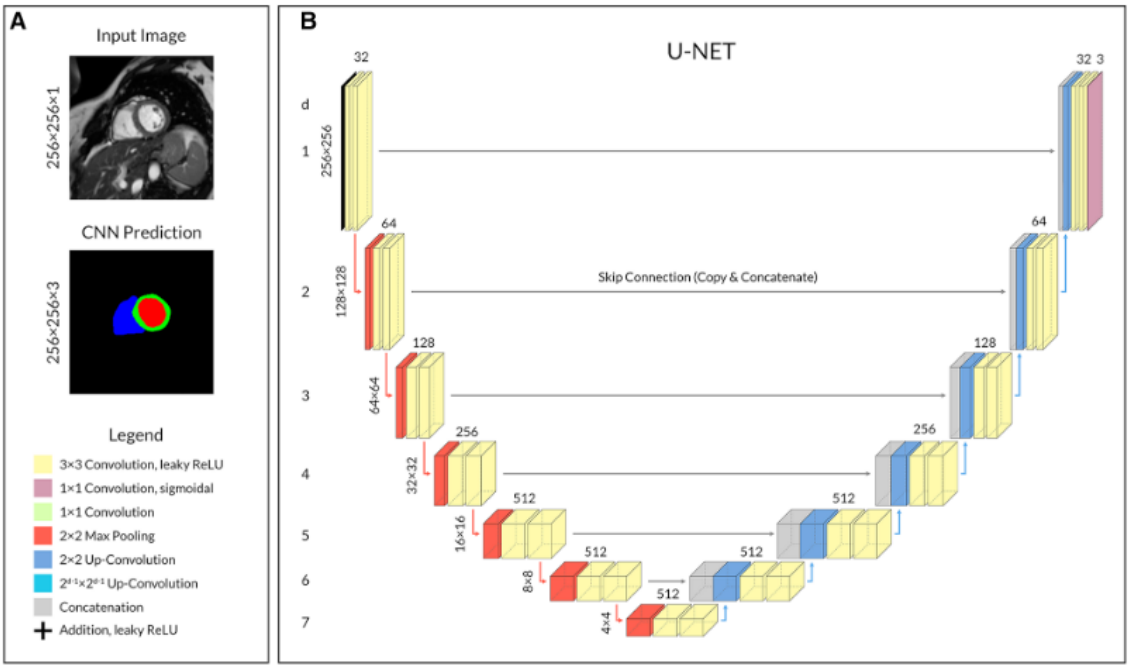

Figure 1 – U-Net Architecture
Illustration taken from “Multilevel comparison of deep learning models for function quantification in cardiovascular magnetic resonance: On the redundancy of architectural variations” by Ammann et al. [2] (DOI 10.3389/fcvm.2023.1118499)
Caption: (A) Network input and output example with corresponding shape. Legend for operational blocks. (B) U-Net with symmetrical encoder and decoder architecture.

In general, the architecture was built to take an input image, scaled to 256x256 image size. It incrementally downscales the input image, while extracting descriptive features, undil the encoding latent space is reached (lowest 4x4 image representation), from which the first up-convolution is derived.

For the short-axis cine data, three segmentation masks must be solved simultaneously for each cardiac structure (i.e. the right ventricle, the left ventricle, and its myocardium). This leads to the three output channels in Figure 1. For T1 and T2 parametric mapping in short-axis position, one single cardiac structure must be determined (left ventricular myocardium), and one reference point must be determined (where the right ventricle touches the left myocardium). This leads to one out-channel for both networks. For reference point detection the same architecture is used, also the training and prediction pipeline must be slightly altered.

2) Training Pipeline

The training pipeline used in this paper was built in Ammann et al. [2] and is inspired from the data augmentations used in NNU-Net by Isensee et al. [3]. The pipeline uses a z-score image normalization by mean and standard deviation of the input image, followed by a strong augmentation schedule. Other parameters are determined by default, as described in Table 1.

Parameter	Description
**Optimizer**	Adam Optimizer (default settings in Pytorch [4])
**Loss function**	Combination of Dice and Cross Entropy (see below for details)
**Layer Normalization**	Batch normalization with a batch size of 8
**Network Depth**	Depth = number of down-samplings reducing image resolution by 2 in the encoder. The network depth is determined such that the image resolution of the encoder reaches 4x4.
**Image Normalization**	Z-score normalization is applied to each image, with image mean and standard deviation
**Augmentations**	Rotation, scaling, translation, shearing, average pooling, gaussian noise, gaussian blurring, gamma correction, contrast enhancement
**Post processing**	Segmentation U-Net: select the largest connected component, removing all other (stray) pixels<br>Reference point detection U-Net: select the maximal intensity in the output mask as the reference point position

The loss functions are customized for the segmentation task (SegU-Net) and the reference point detection task (RefU-Net), respectively. The SegU-Net is trained to predict a binary segmentation mask, such that the Dice and Cross Entropy can be employed as a combined loss function. The RefU-Net on the other hand is trained to predict a heatmap centered around the reference point. To accomplish this, the cross entropy is neglected as it is specialized at categorical class prediction, rather than the prediction of continuous functions. The Dice coefficient is the sole loss function.

References

1. Ronneberger O, Fischer P, Brox T. U-Net: Convolutional Networks for Biomedical Image Segmentation. In: Navab N, Hornegger J, Wells WM, Frangi AF, editors. Medical Image Computing and Computer-Assisted Intervention – MICCAI 2015. Cham: Springer International Publishing; 2015. p. 234–41. (Lecture Notes in Computer Science).

2. Ammann C, Hadler T, Gröschel J, Kolbitsch C, Schulz-Menger J. Multilevel comparison of deep learning models for function quantification in cardiovascular magnetic resonance: On the redundancy of architectural variations. Frontiers in Cardiovascular Medicine [Internet]. 2023 [cited 2023 Jun 5];10. Available from: https://www.frontiersin.org/articles/10.3389/fcvm.2023.1118499

3. Isensee F, Jaeger PF, Kohl SAA, Petersen J, Maier-Hein KH. nnU-Net: a self-configuring method for deep learning-based biomedical image segmentation. Nat Methods. 2021 Feb;18(2):203–11.

4. Paszke A, Gross S, Massa F, Lerer A, Bradbury J, Chanan G, Killeen T, Lin Z, Gimelshein N, Antiga L, Desmaison A, Köpf A, Yang E, DeVito Z, Raison M, Tejani A, Chilamkurthy S, Steiner B, Fang L, Bai J, Chintala S. PyTorch: An Imperative Style, High-Performance Deep Learning Library [Internet]. arXiv; 2019 [cited 2025 Jan 16]. Available from: http://arxiv.org/abs/1912.01703
