# Supplementary material for: Verity plots: A novel method of visualizing reliability assessments of artificial intelligence methods in quantitative cardiovascular magnetic resonance
Source: PLoS One. 2025 May 16;20(5):e0323371. doi: 10.1371/journal.pone.0323371 (PMC12084058; doi:10.1371/journal.pone.0323371)
Supplement: Attachment — Submitted filename: Peer Review- AI and Cardiovascular.docx [file pone.0323371.s007.docx]

|  | Peer Review – Luqman Abass |
| --- | --- |
| Manuscript Number: | PONE-D-25-01126 |
| Full Title: | Verity Plots – A Novel Method of Visualizing Reliability Assessments of Artificial Intelligence Methods in Quantitative Cardiovascular Magnetic Resonance |
| Short Title: | Verity Plots – A Novel Method of Visualizing Reliability Assessments of Artificial Intelligence Methods |
| Originality and Novelty | The study presents original research by introducing a novel visualization method called "Verity Plots" for assessing the reliability of AI methods in quantitative cardiovascular magnetic resonance (CMR). This approach appears to be a new contribution to the field, addressing the need for better tools to communicate reliability and agreement in AI-based quantification. |
| Methodology and Technical Standards | The research methodology is described in detail, demonstrating a high technical standard:   1. The authors clearly define the statistical foundations for their Verity Plots, including tolerance ranges, confidence intervals, and agreement criteria. 2. The study employs well-established AI methods (U-Net) for cardiac image segmentation and parametric mapping, trained on substantial datasets (150 cases for short-axis cine and 224 cases for parametric mapping). 3. The evaluation process is rigorous, with the AI methods tested on independent datasets (318 cases for short-axis cine and 59 cases for parametric mapping) |
| Results and Conclusions | The manuscript presents the results of the Verity Plots' application to various AI methods in CMR. The conclusions appear to be supported by the data presented:   1. Verity Plots provide insights into statistical properties, outlier detection, and parametric test assumptions. 2. The plots offer a framework for determining the acceptability of AI-expert bias and variance. 3. The authors claim that Verity Plots outperform traditional visualization methods like Correlation, Box, and Bland-Altman plots in communicating reliability and agreement.   However, a more detailed presentation of the comparative results between Verity Plots and traditional methods would strengthen these conclusions. |
| Presentation and Language | The manuscript is well-structured and written in standard English. The use of clear definitions, figures, and explanations enhances the intelligibility of the complex statistical concepts presented |
| Ethical Standards and Research Integrity | While the manuscript does not explicitly mention ethical approval, the research appears to adhere to appropriate standards for data analysis and AI development in medical imaging. However, it would be beneficial to include a statement on ethical approval for the use of patient data in training and testing the AI models |
| Reporting Guidelines and Data Availability | The manuscript follows a logical structure typical of scientific papers in the field. However, it would be improved by:   1. Including a clear statement on data availability. 2. Providing more details on the AI model architectures and training procedures. 3. Discussing any limitations of the Verity Plots approach |
| Recommendations | While the manuscript presents a novel and potentially valuable contribution to the field of AI in CMR, I recommend the following revisions:   1. Provide more detailed comparative results between Verity Plots and traditional methods. 2. Include a statement on ethical approval for the use of patient data. 3. Add a clear data availability statement. 4. Discuss potential limitations of the Verity Plots approach. 5. Provide more details on AI model architectures and training procedures. |
