# Supplementary material for: Verity plots: A novel method of visualizing reliability assessments of artificial intelligence methods in quantitative cardiovascular magnetic resonance
Source: PLoS One. 2025 May 16;20(5):e0323371. doi: 10.1371/journal.pone.0323371 (PMC12084058; doi:10.1371/journal.pone.0323371)
Supplement: Attachment — Submitted filename: Response to Reviewers.docx [file pone.0323371.s009.docx]

PONE-D-25-01126

Verity Plots – A Novel Method of Visualizing Reliability Assessments of Artificial Intelligence Methods in Quantitative Cardiovascular Magnetic Resonance

PLOS ONE

Dear Dr. Hadler

Thank you for submitting your manuscript to PLOS ONE. After careful consideration, we feel that it has merit but does not fully meet PLOS ONE’s publication criteria as it currently stands. Therefore, we invite you to submit a revised version of the manuscript
that addresses the points raised during the review process.

Please submit your revised manuscript by Apr 09 2025 11:59PM. If you will need more time than this to complete your revisions, please reply to this message or contact the journal office at [plosone@plos.org](mailto:plosone@plos.org). When you're ready to submit your revision, log on to https://www.editorialmanager.com/pone/ and select the 'Submissions Needing Revision' folder to locate your manuscript file.

Please include the following items when submitting your revised manuscript:

- A rebuttal letter that responds to each point raised by the academic editor and reviewer(s). You should upload this letter as a separate file labeled 'Response to Reviewers'.
- A marked-up copy of your manuscript that highlights changes made to the original version. You should upload this as a separate file labeled 'Revised Manuscript with Track Changes'.
- An unmarked version of your revised paper without tracked changes. You should upload this as a separate file labeled 'Manuscript'.

If you would like to make changes to your financial disclosure, please include your updated statement in your cover letter. Guidelines for resubmitting your figure files are available below the reviewer comments at the end of this letter.

If applicable, we recommend that you deposit your laboratory protocols in protocols.io to enhance the reproducibility of your results. Protocols.io assigns your protocol its own identifier (DOI) so that it can be cited independently in the future. For instructions see: [https://journals.plos.org/plosone/s/submission-guidelines#loc-laboratory-protocols](https://track.editorialmanager.com/CL0/https:%2F%2Fjournals.plos.org%2Fplosone%2Fs%2Fsubmission-guidelines%23loc-laboratory-protocols/1/010f0195363e2394-20643602-63af-46a1-b43b-1bd829d6affa-000000/wLwz6VKiDdyql2UpOaC_SVrLCf3nWYjFEUexwv265Ds=199). Additionally, PLOS ONE offers an option for publishing peer-reviewed Lab Protocol articles, which describe protocols hosted on protocols.io. Read more information on sharing protocols at [https://plos.org/protocols?utm_medium=editorial-email&utm_source=authorletters&utm_campaign=protocols](https://track.editorialmanager.com/CL0/https:%2F%2Fplos.org%2Fprotocols%3Futm_medium=editorial-email%26utm_source=authorletters%26utm_campaign=protocols/1/010f0195363e2394-20643602-63af-46a1-b43b-1bd829d6affa-000000/LljwcKlZks5HRvjCpnQrvC9zv1ngEmVazAylcLWUQ8Y=199).

We look forward to receiving your revised manuscript.

Kind regards,

Haipeng Liu

Academic Editor

PLOS ONE

Journal requirements:

When submitting your revision, we need you to address these additional requirements.

1. Please ensure that your manuscript meets PLOS ONE's style requirements, including those for file naming. The PLOS ONE style templates can be found at

https://journals.plos.org/plosone/s/file?id=wjVg/PLOSOne_formatting_sample_main_body.pdf and

[https://journals.plos.org/plosone/s/file?id=ba62/PLOSOne_formatting_sample_title_authors_affiliations.pdf](https://track.editorialmanager.com/CL0/https:%2F%2Fjournals.plos.org%2Fplosone%2Fs%2Ffile%3Fid=ba62%2FPLOSOne_formatting_sample_title_authors_affiliations.pdf/1/010f0195363e2394-20643602-63af-46a1-b43b-1bd829d6affa-000000/7ee24pqjqyFYzqQXOQolz7k915xRYQ-Ipm8E4wmTWR4=199)

2. Please provide a complete Data Availability Statement in the submission form, ensuring you include all necessary access information or a reason for why you are unable to make your data freely accessible. If your research concerns only data provided within your submission, please write "All data are in the manuscript and/or supporting information files" as your Data Availability Statement.

3. Please include a copy of Table 2 which you refer to in your text on page 14.

Additional Editor Comments (if provided):

Answer: Dear Editor,

we are grateful for the thorough reading of our manuscript, as well as the opportunity to resubmit our manuscript. We believe that the manuscript’s precision and exposition has benefitted significantly from the comments and recommendations:

- We adjusted the manuscript to better fit the PLOS ONE styles:
  - The supplemental material was renamed to fit the PLOS ONE style
  - A Supporting Information section was added below the references for the supplemental material
  - All main sections were made “Heading 1” in Word
- We amended the data availability statement in the submission form
- We uploaded two versions of the revised manuscript, one w/ highlights and tracked changes as well as one w/o
- We removed reference to Table 2 from the manuscript. The reference was accidental. We apologize for this mistake
  - As you will read below, a new table is added to the manuscript for clarification, now called Table 2

The first reviewer’s comments were sent as a separate document, the document – with our responses – is appended below. We explain changes to the manuscript within this response to the reviewers and integrate them into the manuscript, marked in yellow highlight.

In Supporting Information we added:

# Supporting Information

**S1_Fig. Short-Axis Cine Clinical Parameter Differences for Artificial Intelligence Methods.** For all four AI methods, and for cases from all available sites, Condensed Verity Plots are visualized to communicate AI agreement with expert.

**S2_Fig. Short-Axis Cine Clinical Parameter Differences for Artificial Intelligence Methods – Single Site.** For all four AI methods, and for cases from one site, Condensed Verity Plots are visualized to communicate AI agreement with expert.

**S3_Fig. T1 Mapping Clinical Parameter Differences for Artificial Intelligence Methods.** For all four AI methods, and for cases from all available sites, Condensed Verity Plots are visualized to communicate AI agreement with expert.

**S4_Fig. T2 Mapping Clinical Parameter Differences for Artificial Intelligence Methods.** For all four AI methods, and for cases from all available sites, Condensed Verity Plots are visualized to communicate AI agreement with expert.

**S1_File. AI Architecture and Training Pipeline.** Describes the U-Net architecture details employed for training, as well as details of the training procedures, including image normalization and augmentation strategies.

[Note: HTML markup is below. Please do not edit.]

Reviewers' comments:

Reviewer's Responses to Questions

**Comments to the Author**

1. Is the manuscript technically sound, and do the data support the conclusions?

The manuscript must describe a technically sound piece of scientific research with data that supports the conclusions. Experiments must have been conducted rigorously, with appropriate controls, replication, and sample sizes. The conclusions must be drawn appropriately based on the data presented.

Reviewer #1: Yes

Reviewer #2: Partly

Answer: We thank the reviewers for this assessment. We hope that the second reviewer’s reservations are addressed with the alterations below and if they are not, we will do our best to clarify them.

2. Has the statistical analysis been performed appropriately and rigorously?

Reviewer #1: Yes

Reviewer #2: N/A

Answer: Thank you. This is a methods paper focussed on the statistical plot rather than the statistical methods themselves. However, as confidence and tolerance intervals apply to normally distributed data, they require a sample size of N≥30 to be appropriate, which we respected within this proof-of-concept analysis. Throughout the manuscript we aspire to respect all necessary statistical conditions and point out in which circumstances the plot would be used inappropriately. We add the following sentence to “Statistical Test” in Methods :

Parametric tests require normally distributed data, and a sample size of N≥30.

3. Have the authors made all data underlying the findings in their manuscript fully available?

The [PLOS Data policy](http://track.editorialmanager.com/CL0/http:%2F%2Fwww.plosone.org%2Fstatic%2Fpolicies.action%23sharing/1/010f0195363e2394-20643602-63af-46a1-b43b-1bd829d6affa-000000/E0C5P_b5lrvNHrbbcEWR0i-gq2XoPxwLRtsMs5FeKQc=199) requires authors to make all data underlying the findings described in their manuscript fully available without restriction, with rare exception (please refer to the Data Availability Statement in the manuscript PDF file). The data should be provided as part of the manuscript or its supporting information, or deposited to a public repository. For example, in addition to summary statistics, the data points behind means, medians and variance measures should be available. If there are restrictions on publicly sharing data—e.g. participant privacy or use of data from a third party—those must be specified.

Reviewer #1: Yes

Reviewer #2: Yes

Answer: Thanks.

4. Is the manuscript presented in an intelligible fashion and written in standard English?

PLOS ONE does not copyedit accepted manuscripts, so the language in submitted articles must be clear, correct, and unambiguous. Any typographical or grammatical errors should be corrected at revision, so please note any specific errors here.

Reviewer #1: Yes

Reviewer #2: Yes

Answer: We are grateful for this evaluation.

5. Review Comments to the Author

Please use the space provided to explain your answers to the questions above. You may also include additional comments for the author, including concerns about dual publication, research ethics, or publication ethics. (Please upload your review as an attachment if it exceeds 20,000 characters)

Reviewer #1: Thank you for submitting your manuscript "Verity Plots – A Novel Method of Visualizing Reliability Assessments of Artificial Intelligence Methods in Quantitative Cardiovascular Magnetic Resonance." I have thoroughly reviewed your work and find it to be an innovative contribution to the field of AI in cardiovascular magnetic resonance imaging. The introduction of Verity Plots as a novel visualization method for assessing AI reliability is both timely and valuable

Answer: We are glad the reviewer agrees with the necessity of this kind of statistical test and visualization. We thank the reviewer for the comments in the appended word file, which are addressed below.

Reviewer #2: The aim of this paper” Verity Plots – A Novel Method of Visualizing Reliability Assessments of Artificial Intelligence Methods in Quantitative Cardiovascular Magnetic Resonance” is to establish a statistical plot that communicates the reliability of quantitative parameters in CMR by visualizing clear criteria for agreement in addition to descriptive statistical properties. To this end, first, a statistical test is designed to decide evaluation agreement based on bias and variance tolerance ranges. Second, a novel statistical plot is established that combines descriptive statistical properties with agreement criteria, and third, the plot’s utility is demonstrated for AI assessment.
Good work but still need ...

all comments with the editor

Answer: We thank the reviewer for the assessment, and address the comments as the editor passed them on.

6. PLOS authors have the option to publish the peer review history of their article ([what does this mean?](https://track.editorialmanager.com/CL0/https:%2F%2Fjournals.plos.org%2Fplosone%2Fs%2Feditorial-and-peer-review-process%23loc-peer-review-history/1/010f0195363e2394-20643602-63af-46a1-b43b-1bd829d6affa-000000/1cZNS7DapWB_E3DvjcyubIGIzcn3qn74mSVizBOEQj0=199)). If published, this will include your full peer review and any attached files.

If you choose “no”, your identity will remain anonymous but your review may still be made public.

**Do you want your identity to be public for this peer review?** For information about this choice, including consent withdrawal, please see our [Privacy Policy](https://track.editorialmanager.com/CL0/https:%2F%2Fwww.plos.org%2Fprivacy-policy/1/010f0195363e2394-20643602-63af-46a1-b43b-1bd829d6affa-000000/O93U-32VOnD9kRwTeEG3vqGI8eWGiOXvoJZiWTMImFU=199).

Reviewer #1: **Yes:** Luqman Adewale Abass

Reviewer #2: No

|  | Peer Review – Luqman Abass |
| --- | --- |
| Manuscript Number: | PONE-D-25-01126 |
| Full Title: | Verity Plots – A Novel Method of Visualizing Reliability Assessments of Artificial Intelligence Methods in Quantitative Cardiovascular Magnetic Resonance |
| Short Title: | Verity Plots – A Novel Method of Visualizing Reliability Assessments of Artificial Intelligence Methods |
| Originality and Novelty | The study presents original research by introducing a novel visualization method called "Verity Plots" for assessing the reliability of AI methods in quantitative cardiovascular magnetic resonance (CMR). This approach appears to be a new contribution to the field, addressing the need for better tools to communicate reliability and agreement in AI-based quantification. |
| Methodology and Technical Standards | The research methodology is described in detail, demonstrating a high technical standard:   1. The authors clearly define the statistical foundations for their Verity Plots, including tolerance ranges, confidence intervals, and agreement criteria. 2. The study employs well-established AI methods (U-Net) for cardiac image segmentation and parametric mapping, trained on substantial datasets (150 cases for short-axis cine and 224 cases for parametric mapping). 3. The evaluation process is rigorous, with the AI methods tested on independent datasets (318 cases for short-axis cine and 59 cases for parametric mapping) |
| Results and Conclusions | The manuscript presents the results of the Verity Plots' application to various AI methods in CMR. The conclusions appear to be supported by the data presented:   1. Verity Plots provide insights into statistical properties, outlier detection, and parametric test assumptions. 2. The plots offer a framework for determining the acceptability of AI-expert bias and variance. 3. The authors claim that Verity Plots outperform traditional visualization methods like Correlation, Box, and Bland-Altman plots in communicating reliability and agreement.   However, a more detailed presentation of the comparative results between Verity Plots and traditional methods would strengthen these conclusions. |
| Presentation and Language | The manuscript is well-structured and written in standard English. The use of clear definitions, figures, and explanations enhances the intelligibility of the complex statistical concepts presented |
| Ethical Standards and Research Integrity | While the manuscript does not explicitly mention ethical approval, the research appears to adhere to appropriate standards for data analysis and AI development in medical imaging. However, it would be beneficial to include a statement on ethical approval for the use of patient data in training and testing the AI models |
| Reporting Guidelines and Data Availability | The manuscript follows a logical structure typical of scientific papers in the field. However, it would be improved by:   1. Including a clear statement on data availability. 2. Providing more details on the AI model architectures and training procedures. 3. Discussing any limitations of the Verity Plots approach |
| Recommendations | While the manuscript presents a novel and potentially valuable contribution to the field of AI in CMR, I recommend the following revisions:   1. Provide more detailed comparative results between Verity Plots and traditional methods. 2. Include a statement on ethical approval for the use of patient data. 3. Add a clear data availability statement. 4. Discuss potential limitations of the Verity Plots approach. 5. Provide more details on AI model architectures and training procedures. |

Dear reviewer, dear Luqman Abass,

thank you for taking the time to review our paper and providing this thorough and helpful feedback. In the following we answer your questions and explain how we adjust the manuscript to integrate your feedback. We believe that this greatly improved the manuscript.

1. Provide more detailed comparative results between Verity Plots and traditional Methods

We agree with the reviewer that the comparative results between Verity Plots and traditional visualizations can be strengthened. In Figure 5 the extended Verity plots are plotted side-by-side with Correlation, Box, and Bland-Altman plots. However, the reviewer is correct that the differences are not evaluated in detail, but rather qualitatively within the prior paragraph.

We summarize these differences in a Table, in which plot-capabilities are listed, and add it to Results:

“Table 2: Comparison of Reliability Visualization Methods

| Method | Bias | Bulk | Trend | Outlier | Agreement | Scalable |
| --- | --- | --- | --- | --- | --- | --- |
| Correlation Plot | ✖︎ – does not directly show bias | ✖︎ – only shows overall distribution but not limits for proportion | ✖︎ – trends not reliably visible | ✓ – shows outliers in relation to overall distribution | ✖︎ – not available | ✖︎ – uses x- and y-axis |
| Boxplot | ✓ – shows the median | ✓ – shows quantiles of the data distribution | ✖︎ – cannot track systematic trends | Partially – shows single outliers | ✖︎ – not available | ✓ – only y-axis |
| Bland-Altman Plot | ✓ – shows the mean difference | ✓ – limits of agreement contain 95% of data | ✓ – can reveal trends across x-axis | ✓ – outliers visible as outside limits of agreement | ✖︎ – not available | ✖︎ – uses x- and y-axis |
| Extended Verity Plot | ✓ – shows the mean difference + confidence interval | ✓ – bulk of data inside tolerance intervals | ✓ – can reveal trends across x-axis | ✓ – outliers leave the variance tolerance range | ✓ – shows agreement as reliability plot | ✖︎ – uses x- and y-axis |
| Condensed Verity Plot | ✓ – shows the mean difference + confidence interval | ✓ – bulk of data inside tolerance intervals | ✖︎ – only uses y-axis, x-axis collapsed | ✓ – outliers leave the variance tolerance range | ✓ – shows agreement as reliability plot | ✓ – only y-axis |

A qualitative comparison of Verity Plots and traditional reliability visualization methods (i.e. Correlation, Box, and Bland-Altman plots) across key assessment criteria. Verity Plots offer advantages in detecting trends, outliers, and the concentration of data, while traditional methods have limitations in communicating distribution characteristics or agreement.

Legend: ext.: extended, cond.: condensed”

We add the following to Methods:

The results of this comparison are summarized in a table.

We add the following to Results: “The analysis is systematically summarized in Table 2.”

We further move a significant portion of the descriptive text of the plots below Figure 5 and before Table 2, so that the reader can better follow how support for the claims in Table 2 are gathered in Figure 5.

2. Include a statement on ethical approval for the use of patient data.

We gladly follow the reviewer’s advice, and make the statement on ethical approval clearer, specifically on how it pertains to patient data. In the manuscript in Methods under “Ethics Declarations and Data Access”:

“The use of patient data for this study was approved by the local ethics committee of the Charité Universitätsmedizin Berlin as retrospective study (study ID: EA 1253 21).”

3. Add a clear data availability statement.

We agree that the availability statement should be clearer as the differentiation between MRI data and numerical data is unclear. In Methods under “Ethics Declarations and Data Access” we add:

“All numerical datasets required to reproduce the results are available in the supplemental material.”

Further to clarify that the MRI data (used to train the AI methods) is not available we add:

The MRI datasets cannot be shared due to institutional rules but may be available on special request.

4. Discuss potential limitations of the Verity Plots approach.

We thank the reviewer for this interesting question. Critiquing Verity Plots (extended and condensed) should focus on their utility in understanding data distributions. In order to pre-empt the extended Verity Plot weakness of not scaling well across multiple comparisons, we developed a condensed version as well – which collapses the x-axis to allow for multiple assessments.

Nonetheless, they rely on human interpretation, which may introduce variability in assessment across different users. Future studies should evaluate their usability across a broader range of clinical and technical settings. We add the following to the discussion:

“Verity plots, like all visualization methods, rely on human interpretation, which may introduce variability in assessment across different users. Future studies should evaluate their usability across a broader range of clinical and technical settings.”

However, the statistical agreement test and its accompanying reliability plot are limited to normally distributed data. In order to perform this parametric test, the data must be normally distributed and the sample size must be larger than 30. Confidence and tolerance intervals are difficult to replace with non-parametric versions, but they can be approximated with bootstrapping for median and percentile approximation. However, these methods require larger sample sizes to be stable, which is often infeasible for medical imaging quantitative parameters. We add this limitation to the Discussion:

“As a parametric test, the agreement test communicates whether two QP assessment methods agree if their differences are normally distributed and a sample size larger than 30 is provided. Non-parametric confidence and tolerance intervals for a non-parametric agreement test are possible but would require larger sample sizes, which are often infeasible for QPs based on medical imaging.”

5. Provide more details on AI model architectures and training procedures.

We understand the reviewer’s interest in the AI models – in fact an earlier version of the manuscript focussed more on the AI training procedures. However, when finalizing the manuscript we limited the description in order to focus the reader’s attention on the Verity Plots, and using the AI methods exclusively as tools to illustrate the Verity Plot’s utility. However, we gladly expand on the AI model architectures and their training:

The AI architectures are convolutional neural networks (CNN), implemented as UNets, as originally described by Ronneberger et al. [1]. The training procedure builds on the NNUNet approach published by Isensee et al., which currently provides one of the state-of-the-art approaches to biomedical image segmentation challenges. Isensee’s team makes the argument that CNN architectures are overrated and no significant architectural improvements have been made since the original UNet [2]. Instead, Isensee focusses on an automated optimization of the CNN training setup, including network parameters (such as the learning rate, loss function, optimizer, image resampling strategy or network depth).

We selected the UNet architecture based on a similar reasoning investigated in previous work on an architecture comparison at CMR post-processing [3]. Here, we implemented and tested three popular segmentation CNNs: the UNet, the fully convolutional network (FCN) [4], and the MultiResUNet [5]. The UNet architecture used in this paper for the segmentation tasks, as well as the reference point detection tasks, have the same architecture:

Illustration taken from “Multilevel comparison of deep learning models for function quantification in cardiovascular magnetic resonance: On the redundancy of architectural variations” by Ammann et al. [3] (DOI 10.3389/fcvm.2023.1118499)

Caption: (A) Network input and output example with corresponding shape. Legend for operational blocks. (B) U-Net with symmetrical encoder and decoder architecture.

The training procedure is also built similarly to the one in the architecture comparison paper, with the following parameters:

| Parameter | Description |
| --- | --- |
| Optimizer | Adam Optimizer (default settings in Pytorch) |
| Loss function | Combination of Dice and Cross Entropy (see below for details) |
| Layer Normalization | Batch normalization with a batch size of 8 |
| Network Depth | Depth = number of down-samplings reducing image resolution by 2 in the encoder. The network depth is determined such that the image resolution of the encoder reaches 4x4. |
| Image Normalization | Z-score normalization is applied to each image, with image mean and standard deviation |
| Augmentations | Rotation, scaling, translation, shearing, average pooling, gaussian noise, gaussian blurring, gamma correction, contrast enhancement |
| Post processing | Segmentation UNet: select the largest connected component, removing all other (stray) pixels  Reference point detection UNet: select the maximal intensity in the output mask as the reference point position |

Loss Functions of Segmentation and Reference Point U-Nets:

The difference between the segmentation and the reference point detection networks (S-UNet, RP-UNet) is limited to the S-UNet predicts a binary segmentation mask, whereas the RP-UNet predicts a heatmap, centred around the reference point. This has an implication for the loss functions used during training: the S-UNet is trained with the Dice and the Cross-Entropy, whereas the RP-UNet is trained with Dice exclusively.

We considered adding these comments to Methods in the manuscript, but we believe this may refocus the reader on the architectures and AI training, which is relevant but not the centre of this paper. Instead, we recommend adding this information as supplemental material to the paper. To Methods, under “Training of Artificial Intelligence Methods”, we add:

“For an in-depth description of the employed U-Net architecture and training procedure see the supplemental material (S1_File.pdf).”

(The pdf was uploaded with this reviewer response)

**References**

1.	Ronneberger O, Fischer P, Brox T. U-Net: Convolutional Networks for Biomedical Image Segmentation. arXiv:150504597 [cs] [Internet]. 2015 May 18 [cited 2021 Dec 11]; Available from: http://arxiv.org/abs/1505.04597

2.	Isensee F, Jaeger PF, Kohl SAA, Petersen J, Maier-Hein KH. nnU-Net: a self-configuring method for deep learning-based biomedical image segmentation. Nat Methods. 2021 Feb;18(2):203–11.

3.	Ammann C, Hadler T, Gröschel J, Kolbitsch C, Schulz-Menger J. Multilevel comparison of deep learning models for function quantification in cardiovascular magnetic resonance: On the redundancy of architectural variations. Frontiers in Cardiovascular Medicine [Internet]. 2023 [cited 2023 Jun 5];10. Available from: https://www.frontiersin.org/articles/10.3389/fcvm.2023.1118499

4.	Long J, Shelhamer E, Darrell T. Fully Convolutional Networks for Semantic Segmentation [Internet]. arXiv; 2015 [cited 2022 Jun 2]. Available from: http://arxiv.org/abs/1411.4038

5.	Ibtehaz N, Rahman MS. MultiResUNet : Rethinking the U-Net Architecture for Multimodal Biomedical Image Segmentation. Neural Networks. 2020 Jan;121:74–87.
